# Untangling Ageism: Insights From Diverse Perspectives, Methods of Inquiry, and Contexts

**DOI:** 10.1093/geroni/igaf122.914

**Published:** 2025-12-31

**Authors:** Liat Ayalon

## Abstract

Ageism, defined as stereotypes, prejudices, and discrimination, has been studied for over five decades now. Yet, it is still more prevalent than the other big “isms” such as racism or sexism but is less recognized than other “isms.” In this symposium, we present research conducted in various socio-cultural contexts including Canada, China, Germany, Israel, and the United States to highlight the complexity and multi-faceted nature of ageism. Levy and Martin present research from the Health and Retirement Survey showing the importance of positive views on aging in reversing and preventing the development of mild cognitive impairment. Xi and colleagues highlight the paradox of digital ageism in two different cultural contexts: the United States and China, demonstrating differential age and culture effects of digital ageism on technology use . Ayalon and colleagues examine shifts in society and their potential effects on ageism. They demonstrate a reduction in ageism following the Sward of Iron war in Israel. de Paula Couto & Rothermund examine the interaction between chronological age and disengagement norms, showing a reduction in the reports of age discrimination in those older persons who are more likely to hold disengagement norms. Finally, Gans & Chasteen discuss intersectional identities in relation to prescriptive norms, demonstrating that older White men are the ones most expected to step aside for younger generations and most negatively judged as moral violators if they fail to comply with prescriptive age norms. The studies highlight the importance of context and the distinction between prescriptive and descriptive age norms.

